# Barriers and facilitators to implementing advance care planning in naïve contexts - where to look when plowing new terrain?

**DOI:** 10.1186/s12877-023-04060-4

**Published:** 2023-06-23

**Authors:** Siri Færden Westbye, Siri Rostoft, Maria Romøren, Lisbeth Thoresen, Astrid Klopstad Wahl, Reidar Pedersen

**Affiliations:** 1 Centre for Medical Ethics, Institute of Health and Society, Faculty of Medicine, University of Oslo, Postboks 1130, Blindern, 0318 Oslo, Norway; 2 Institute for Clinical Medicine, Faculty of Medicine, University of Oslo, Oslo University Hospital, Oslo, Norway; 3 Department for Interdisciplinary Health Sciences, Faculty of Medicine, Institute of Health and Society, University of Oslo, Oslo, Norway

**Keywords:** ACP, Elderly, Hospitals, Primary care, Health services, Autonomy

## Abstract

**Background:**

Advance care planning (ACP) is a way of applying modern medicine to the principle of patient autonomy and ensuring that patients receive medical care that is consistent with their values, goals and preferences. Robust evidence supports the benefits of ACP, but it remains an underutilized resource in most countries. This paper goes from the naïve point of view, and seeks to identify the barriers and facilitators to implementation in unfamiliarized contexts and in a whole system approach involving the clinical, institutional and policy level to improve the implementation of ACP.

**Methods:**

Qualitative interviews were chosen to enable an explorative, flexible design. Qualitative interviews were conducted with 40 health care professionals and chief physicians in hospitals and in municipalities. The thematic analysis was done following Braun and Clarke’s strategy for thematic analysis.

**Results:**

The main reported barriers were the lack of time and space, a lack of culture and leadership legitimizing ACP, lack of common communication systems, and unclear responsibility about who should initiate, resulting in missed opportunities and overtreatment. Policy development, public and professional education, and standardization of documentation were reported as key to facilitate ACP and build trust across the health care system.

**Conclusions:**

Progressively changing the education of health professionals and the clinical culture are major efforts that need to be tackled to implement ACP in unfamiliarized contexts, particularly in contexts where patient's wishes are not legally binding. This will need to be tackled through rectifying the misconception that ACP is only about death, and providing practical training for health professionals, as well as developing policies and legislation on how to include patients and caregivers in the planning of care.

**Supplementary Information:**

The online version contains supplementary material available at 10.1186/s12877-023-04060-4.

## Background

The possibilities of prolonging the life of severely ill older patients are increasing public expectations and costs of medical care, making decisions about terminating care more challenging [1, 2]. This raises new ethical questions, related to the main ethical principles of autonomy, beneficence, non-maleficence, and justice [3]. Advance care planning (ACP) can be a way of dealing with these ethical questions and balance ethical principles [4]. ACP is defined as a process that supports the patient in sharing their personal values, life goals and preferences regarding future health care and treatment [5]. The goal is to ensure that people receive health care that is consistent with their values, goals and preferences during serious and chronic illness [5], but also reducing overtreatment and undertreatment [6].

### Evidence and knowledge gap

Firstly, there has been significant shifts in the concept of ACP. Originally ACP was conceptualized mainly as the completion of an advance directive (AD) where treatment choices were exchanged between patient and physician in a more legal transactional approach [7]. However more recently, international ACP experts have agreed on that ACP should be understood as a communicative process [5] and that it would be important to broaden the concept of ACP to not just be about the end of life [5]. This project is based upon these new conceptions of ACP.

Secondly, there is a knowledge gap in the translation of the evidence of ACP into practice. Although the effectiveness of ACP has been debated [8] and reviews from 2018, 2021 and 2022 found limited evidence that ACP improves the chances of goal-concordant care [9–11], by large the recent summary of evidence supports the potential of ACP to support the involvement of older patients and their caregivers and improve communication. The 2021 systematic review focusing only on randomized control trials (*n* = 69, of which 94% rated high quality) found that 88% of studies showed positive impact upon patient-surrogate/clinician congruence. They found that 100% of studies had a positive impact on patients/surrogate/clinician satisfaction with communication, and 75% had a positive impact on surrogate satisfaction with patients' care [10]. The newest and largest review to date [11] focusing only on randomized controlled trials (*n* = 132, of which 64% rated high quality) also found consistent evidence that ACP interventions improves quality of patient–physician communication (68%), preference for comfort care (70%), decisional conflict (64%) and patient-caregiver congruence in preference (82%) [11]. Despite this, ACP remains an underutilized resource in clinical practice [12–17]. The question is why?

Available evidence suggests that there are multiple barriers for ACP (Additional file 1) but this evidence focuses almost exclusively on end of life. This compartmentalization in the evidence may overlook critical contextual factors that influence ACP implementation [9] if ACP is to be broadened as concept [5]. Complicating the interpretation of evidence furthermore, is that the term end of life is inconsistently defined, although a common understanding is that it refers to the final hours, days, weeks, or months in a person’s life [18].

Sharp (2013) [12] and Lund (2015) [19] published the first systematic reviews on implementation problems, as well as barriers and facilitators, but these reviews were limited to end of life, as is Fien and colleagues recent scoping review (2021) [14]. Jimenez (2018) [9] and Combes (2019) [20] are the first reviews to adopt the concept of ACP that not necessarily translates into end of life, but more research is needed within this broader concept [9]. Available evidence also lacks a systemic approach in the implementation of ACP. Jimenez and Combes stress the need for a “system-wide” or a “whole system-strategic approach”. Systems theory is a conceptual framework based on the principle that the component parts of a system can best be understood in the context of the relationships with each other and with other systems, rather than in isolation [21]. The principles of systems theory have been applied across fields and disciplines from natural sciences, business management to child psychology, particularly Bronfenbrenner's ecological systems theory theorizing four layers of interrelated systems. Bronfenbrenner called these layers the a) microsystem (the system closest to the client, or the patient or child, depending on the field of research); b) the mesosystem (where microsystems interact); c) the exosystem (external work environment); and d) the macrosystem (larger socio-cultural context). It is this concept of interacting systems and the importance of shared problem-solving within and between these systems that provide a potentially powerful model for developing and delivering interventions in clinical contexts [21]. In Jimenez summary of evidence on ACP, a ‘‘whole system strategic approach’’ means to see ACP as an interconnected set of elements relying on each other, instead of focusing separately on its individual components [9]. Combes describes the “system-wide” approach as something that occurs over time, rather than as a one-off event. To enable this, professionals need support from engaged chief physicians within their organizations and the wider system [20].

The systems perspective also relates to the problem that there is no unified program for ACP. Indeed there is a great variability in the way ACP is approached and conceptualized in the literature [9]. Legally and politically there is also a wide diversity of approaches to ACP, even within countries [9]. In Norway, where this research has been conducted, the legal and political status of ACP is challenging. Legally, respect for autonomy is operationalized through the doctrine of informed consent as is the case for most modern care health laws. However, in situations where the patient is considered not competent, the doctors’ always have the final say [22]. Furthermore the lack of substitute decision maker in Norway [22] is also likely to have a large impact on ACP. These contextual factors are of great importance for the interpretation of evidence, since the majority of studies have been conducted in the US [9–11].

Politically there has been some interest for ACP in Norway, and ACP has been enshrined in national recommendations [23, 24], but is not consistently used due to the implementation problem of ACP and the lack of a unified approach. Albeit there have been major efforts to agree on a definition, recommendations and outcomes [5, 25, 26], there is still a knowledge gap on how to implement ACP as a program throughout the health care services, particularly in a context where ACP is not known to health care professionals, nor the public. Notably Jimenez and Combes support the need for making a unified program that can be used as a starting point for professionals, organizations and policymakers [9, 20]. Hence, there is a need for further studies evaluating the impact of ACP for different populations, settings, and contexts to unleash ACP's full potential [9].

### Contribution to the field

This study is a sub-study part of a large Norwegian project “Implementing ACP—A Cluster Randomized Controlled Study”. This multicenter project will test whether implementation of ACP actually improves outcomes for patients, family, and services in real-life services.

The most important contribution of this sub-study is to provide knowledge about the perceived barriers and facilitators in a clinical environment where there has been no systematic implementation of ACP. The findings are intended to inform the large implementation project.

Secondly, the contribution of this sub-study is to provide knowledge from a whole system perspective. Because this is a complex intervention where cooperation across levels is a prerequisite, we have sought information about users’ needs at all levels. This is the first study to explore the barriers and facilitators at the clinical, organizational, and policy level and in the hospital and community setting, as well as the cooperation between these settings.

Hence this is a contribution to how ACP can be better implemented across settings in a whole system approach and to what can be used as a unified, starting program for ACP. Finally, this is one of few studies not limiting the concept of ACP to end of life.

The main research question for this paper is therefore what are the barriers and facilitators for implementing ACP for severely ill older persons in a whole system approach from the perspective of health personnel and chief physicians in hospitals and community services. The overall aim is to explore what characterizes good ACP and good implementation strategies that can be shared in contexts that are naïve to ACP.

## Methods/design

### Design

The COREQ guidelines have been used to report this qualitative research [27] (Please see Additional file 1). Interviews were chosen to enable an explorative, flexible design [28] to identify what health care professionals and chief physicians perceive as barriers and facilitators for implementing ACP. The methodology that guided the qualitative approach was a hermeneutical approach, rooted in Gadamers’ philosophical hermeneutics. This means that we are aware that the researcher’s preunderstanding of the issue at stake, in this case ACP, informs the different stages of the research process. In developing new insights and knowledge on ACP, the researcher’s as well as the participants’ preunderstandings is involved. As such, hermeneutics is a process of co-creation between the researcher and participant, in which the very production of meaning and knowledge occurs through a hermeneutic circle of listening and dialogue, as well as reflective writing and interpretations [29]. The interview guides were therefore semi structured to provide some consistency in topics, while allowing flexibility, so that there was room to explore new topics. The interview guides were internally validated within the research team with particular respect to allowing this flexibility before conducting the interviews. The researcher’s background as a doctor has been made clear through keeping a log and discussing positionality during data collection and analysis. This helped clarify bias regarding whether ACP should be done by doctors, and taking care to also present results counter to promoting ACP, particularly participants’ critical perspective to ACPs utility and effectiveness. Thoroughly considering foundational questions regarding timing and the setting for ACP, possibly intruding in older persons’ lives, and acknowledging the work health personnel are already doing, have also been important discussions. The authors only had connection to the field of ACP through research, apart from a member of the research team in the larger project that had practical experience with ACP.

### Participants

The sampling strategy was a combination of a purposeful and snowballing method. The purposeful method was due to the professional networks known to the researchers. The background characteristics required were experience with discussions with similarities to ACP. Only three participants had practiced ACP. The hospital wards were first selected to represent patient groups for whom ACP was relevant (COPD patients/respiratory medicine units, acutely ill older patients living with frailty/ geriatric units), as well as geographical placement (rural/urban). During the planning of the large project that will follow this sub-study, we decided to implement only in geriatric units because the interest and motivation was considerable in these units. We considered it justifiable since this could provide good grounds for further implementing ACP nationally and to larger parts of the services. Therefore we continued to interview in geriatric units, to seek knowledge from the context where we will be implementing. The participants were partly junior and senior physicians and nurses in hospitals, and the GPs were moderately to very experienced (range: 11–40 years’ experience) to seek variation in viewpoints. We recruited participants from hospitals through the chief physician of each respective ward. Nurses and doctors were invited to participate. All the participants agreed to take part in the interviews. A key person would be a person with particular knowledge and experience with the implementation of ACP. It was hard to find anyone with this experience. An overview of the participants’ characteristics can be seen in Table 1.

**Table 1:** Characteristics of participants

**Group interviews** **1 respiratory ward, 3 geriatric wards**		**Individual interviews**		
		**Chief physicians (4)**	**GPs (8)**	**Key persons (3)**
Doctors	20	4		
Nurses	5			1 (palliative nurse)
Senior^*^	20	4	6	3
Junior^**^	5		2	
Prof. background				
Geriatrician	12	3		2
Respiratory physician	3	1		
Internist	2			
Gastroentorologist	1			
Neurologist	2			
Palliative care physician	1	1	2	
Female	18	1	2	1
Male	7	3	6	2

^*^Senior: specialist GP/attending or ward leader nurse

^**^Junior: resident, GP or ward nurse

### Data collection

Nineteen qualitative interviews were conducted in total. The total number of participants was 40. The interviews consisted of four group interviews with doctors and nurses in the selected hospital wards, and seven individual interviews with general practitioners (GPs), five chief physicians in communities and hospitals, as well as three individual interviews with key persons. Group interviews were chosen for feasibility among busy hospital staff, and to allow discussions. The groups were heterogeneous with regards to the numbers of attending participants, and that mostly doctors participated. The first interview consisted of four doctors and one nurse. The second interview consisted of two doctors and two nurses, the third interview of two doctors and two nurses, and the last of ten doctors and two nurses. In the research process the material was expanded to include logs from the planning phase of the larger project. Logs included quotes from chief physicians during recruitment meetings where key issues such as barriers, timing, setting and patient population were discussed to achieve saturation in the data.

### Procedure

Following on the framework of hermeneutics and particularly the process of co-creation between the researcher and participant, the interviews had the form of an epistemological collaboration [28]. The researcher tried to help participants articulate in dialogue what they perceived as barriers and facilitators. All the interviews were conducted by a female researcher, SFW. SFW presented herself as a PhD student and medical doctor (MD), where two of the participants were prior known to her. Reasons for doing the research (better involvement of older patients, developing ACP in our community), the assumption that this is unfamiliar practice, as well as the interest in developing ACP in Norway were reasons conveyed to participants prior to interviewing. All of the interviews were conducted in Norwegian. The group interviews were done in quiet shielded meetings rooms in the hospital. However, physicians would have their pager on them. In the first interview one doctor left during the interview, and in the fourth interview two doctors left during the interview. Their respective chief physicians were interviewed separately in the same room, and stayed throughout the interview. The individual interviews with GPs and municipal chief physician, and key person interviews were done either by phone or zoom. Details on the room, participants’ profession, and who left where noted down. No one else was present besides the participants and researcher. Interviews lasted between 28 and 65 min. All the interviews were conducted and audio recorded between March 2020 and March 2022. Repeat interviews were not carried out, and transcripts were not returned to participants for comments.

### Analysis

SFW underwent training in qualitative research methods and was supervised by a research team entailing one experienced qualitative researcher and nurse (LT), a geriatrician (SR) a philosopher and MD (RP) and a GP (MR). Thematic analysis (TA) of the interviews was done following Braun and Clarke’s 6-step method [30] with particular respect to reflexivity throughout the research process [31]. The first three steps in the analysis were done by SFW under supervision by RP. The first step consisted in transcribing recorded interviews which were transcribed verbatim, in Norwegian, by AGH and JS. Transcriptions checked for accuracy by SFW, reading and re-reading the data, and noting down initial ideas as a way of getting familiar with the data. The second step consisted of coding interesting features of the data in a systematic fashion across the entire data set, collating data relevant to each code. The third step was collating codes into potential themes, gathering all data relevant to each potential theme. The reviewing of these themes (step 4) and refining the specifics of each theme (step 5) were done in collaboration with the supervisors (RP, LT, SR, MR) to make suggestions about further links between themes, categories and concepts. Participants themselves did not provide feedback on the themes. Only the quotes used in the report were translated into English. Nuances in meaning were preserved in translation, by using a native English that read through the quotes, and using Norwegian-English dictionaries for accuracy and spelling.

### Quality

We have tried to achieve transparency in this study. The researcher has made her positioning clear and showed that this could pose as a challenge. Nevertheless, the positioning could also be applied to promote an understanding of what is going on. A concrete example was to have knowledge about locally used languages and vocabularies [28] patients' trajectories, disease management and forms of care available. The different viewpoints were consistent and confirmed by participants from within the different levels in the system, and they are relevant for cultures were the status of ACP is challenging. We believe that the insight is sound and may be portable and useful to other contexts naïve to ACP.

### Research ethics

This research complies with international and national standards and has been performed in accordance with the Declaration of Helsinki. It has been approved by the appropriate national ethics committees. NSD- Norwegian Centre for Research Data, the Data Protection Official for Research, approved the study on February 25 2020. The legal basis for their approval is the Norwegian Personal Data Protection Regulation Act art. 6. NSD’s reference number is 150,740.

Written informed consent was provided by all participants in the interviews and for relevant parts of the log. Participant’s names and corresponding key numbers to the interviews were stored separately at the University of Oslo’s Services for Sensitive Data (TSD). Only employees in the project (SFW, RP, AGH and JS) had access to the data files and duty of confidentiality was taken and signed by the transcription assistants. Professional and ethical judgement was used with regard to how participants were represented especially with regards to differing cultural and political opinions regarding the physician’s role and duty and the patient’s legal and ethical rights and considerations.

## Results

In this study new barriers and facilitators to the implementation of ACP were reported. They are original in terms of being from a context where ACP is new, patients’ wishes are not legally binding and appointing a substitute decision maker does not exist. At the clinical level, new facilitators were to correct the misconception that ACP does harm, or that it is only about death in the training and culture. At the institutional level setting up standards for documenting ACP across levels that are safe was another new facilitator to build trust around ACP. At the policy level, deciding on that health professionals are responsible for initiating ACP and including how to do this this in the education was a new facilitator. Finally developing policies and legislation concerning patient participation and how to involve patients and caregivers in medical and care planning were original facilitators identified at the policy level.

**Fig. 1:**
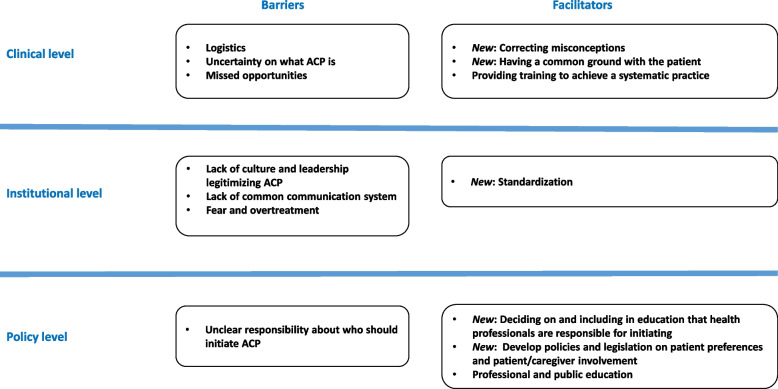
Barriers and facilitators to implementing ACP

All the results will be presented as barriers and facilitators at the clinical, institutional and policy-level (Fig. 1) with overarching themes for each level. Participants’ quotes are used to support the description of each overarching theme.

### Barriers to implementation of ACP

#### Logistics

At the clinical level, the lack of time stood out as a very clear pattern reported by clinicians and chief physicians in communities and hospitals. The lack of single rooms in hospitals was often mentioned as a co-factor, stressing the problem of confidentiality and the problem of making ACP too hastily.

“We are pressured in terms of time and space.” (Senior hospital doctor)

“I don’t have time to document such heavy conversations. I have 15 min per patient.” (GP)

### Uncertainty on what ACP is

Apart from the key persons, all participants were uncertain about what ACP was. The overall pattern was that for now, ACP represents more of an idealistic program known to participants through English speaking literature or heard of sporadically through persons with special interest. When the philosophy of ACP was described to them, all participants recognized the significance and the need for ACP, but were uncertain about when and how to do ACP in a good way. The participants who were doctors, reasoned mostly by saying that ACP disregarded of its intent could hurt the patient inflicting with the physician’s obligation not to harm.

“It must not be the case that [we decide on] that ACP is important, we must have it, and then it can actually do more harm than good [beneficience]. Yes, beneficience was a good word. It can do a lot of damage if the wrong person does it the wrong way. It can be very traumatic.” (Experienced GP).

“It becomes as an opposite to giving hope, it can feel that you turn it around, and […] that you are placing the stone that burdens.”(Experienced GP)

Secondly, there was a preconception that ACP was only about death, or end of life issues.

“You go to the doctor to get well. Not to plan death” (GP)

«It’s hard to avoid the graveyard in a conversation like this» (Chief physician)

### Missed opportunities

An aligned theme were the often missed opportunities to have ACP. Throughout the material there was an uncertainty about who should initiate, and how difficult it would be to bring it up.

“We think the patient will bring it up. But the patient is ill, and the patient is scared, and they meet someone in a uniform.” (Chief physician)

There was also a recurrent hope that someone else would perform the talk.

“You just hope that someone else will take care of that conversation” (GP).

Participants also had an understanding that they were doing ACP, while what they were describing was making decisions for things happening during that hospital stay.

“Often there is much more focus on the purely medical and too little space and time to address what may occupy the patient the most. For instance, how am I going to die, how is it going to be. I think that is very important to talk about, and often it is talked about much too late” (Junior hospital doctor).

“I have little experience of in a way discussing what to do at the next hospital stay, or planning further ahead.” (Senior hospital doctor).

Typically when a discussion about the treatment level would be had, the patient would be far down an illness-trajectory. At that point there would already be a missed opportunity to have ACP for the right reasons, but the scope of the conversation would also be narrowed down to a clarification of treatment. Even more problematic, was the idea that ACP could be used as a way of approaching the topic of limitation of treatment.

“The times I think, at least personally, this conversation is sought is where we have a conception that we should enter a limitation of treatment” (Junior hospital doctor).

### Lack of culture and leadership legitimizing ACP

Uncertainty and lack of knowledge combined with a lack of personal ability made many participants curb their enthusiasm around ACP and have an attitude of “let’s see what happens”. This was a common attitude for both GPs and hospital personnel who were sitting on the fence and waiting for the legitimation of ACP in the leadership and in the culture.

“I think we have to start somewhere. But I think building up the attitudes [towards ACP] is important” (Junior hospital doctor).

“There is more talk about autonomy and participation and things like that. But the threshold for doing it yourself…” (Key person).

### Lack of common communication system

The lack of a joint communication system across hospitals and across levels was an important barrier reported on the institutional level. This had to do with trust in that the information would be interpreted correctly in the other end.

“I experience that I spend an incredible amount of time coordinating cooperation between the various levels in the health service. I am often left with the feeling that this work is meaningless because I do not know how it is documented by the other stakeholder, and I also do not know how what we have agreed upon is followed up” (GP).

“I experience that we to a large extent do not trust or feel that things are not done” (GP).

Examples of this was to document as little concrete as possible, or using the next of kin as a messenger, or giving out the telephone number of the doctor so that he or she could be contacted off hours. Avoidance or fear of giving out the wrong answer when asked about patients’ preferences was also reported.

“Even in nursing homes it is very often that the staff does not know this. When I call and ask, they say «no, no, no [evasive voice] so it is very difficult. The wishes are of course there, well sorted somewhere.” (GP).

### Fear and overtreatment

In many instances the material reflected a pattern of overtreatment, much related to the lack of interaction and communication between levels in the system. Community physicians described this as a “loss of the patient” in emergency situations, and hospitals physicians as the default of overtreatment.

“We spend an awful lot of time every single day figuring out what the right level of treatment is […] and then we overtreat before we clarify the situation. And then we bring in next of kin, and it becomes very difficult, because the situation is acute right, and then you express yourself it in the wrong way and the next of kin think they should decide it, and then they want to overtreat” (Hospital doctor).

“You do it yourself in a way [talking about overtreatment]. It’s easier to try” (Hospital doctor).

Similarly, the anxiety of the next of kin in an emergency situation was an aspect difficult to take into account without ACP, the result often being hospitalization anyway.

“It is hard to handle [for the next of kin] when the patient who is seriously ill gets worse, and then having to deal with it alone, typically at home in the evening, when it happens” (GP).

### Facilitators to implementation of ACP

#### Standardization

Firstly all participants expressed a current matter of fact need for standardization both at the clinical and the institutional level. This would practically mean developing guides, adding templates to the patient electronic record system and standards for communicating ACP. But the participants also acknowledged that templates would never replace the individual approach.

“You must be sensitive to the [laughs briefly] feedback that the patient gives. So that we have a guide that we ask from, but you cannot just run through it” (Experienced GP).

“It is important to see where the patient is at all times. Where is the patient sitting in front of you—and then you have to adapt ACP to that” (Chief physician).

### Having a common ground with the patient

The consideration for the patient’s state and tuning into the patient, relates to the theme of having a “common ground” with the patient explained as having the same realistic image of the patient’s health status. Experienced participants explained this as the facilitating counterpart to hurting or practicing non-maleficence.

“It is very unnatural for people who do not identify with the situation to plan for it. You can say that it hurts anyway. […] Trying to get the patient’s point of view in relation to what they think about their situation and the prognosis is my way into things here. And if they have an understanding that I think is reasonable in relation to what I think is reality, then it is a good starting point.” (GP).

However this could also reflect a lack of knowledge since one of the first steps of ACP is exactly to make sure the patient has sufficient knowledge about their current health, identify possible misconceptions’ and provide the patient with unambiguous information so as to increase their engagement [32].

### Providing training to achieve systematic practice

This theme is closely related to having sufficient training, particularly in communicating with older patients about their psychosocial needs. Both GPs and hospital personnel expressed a need for more systematic training regarding these issues and situations, particularly how to follow up on patients’ emotional hints during a conversation.

“The patient can bring up a vague question and so on, and if you choose, as a doctor…at least I think it is sometimes to protect ourselves, when they bring up what they worry about, then it’s not difficult to skip that part. And then the patient understands that I cannot talk to this doctor about such things”. (Experienced GP).

“I wish I had a tool or a systematic approach to this.” (Junior hospital doctor).

### Policy development

Implementing more training in the educational system was regarded as a facilitator by chief physicians and key person interviews. But they also gave clear statements about how acceptance of the patient’s preferences in policy development ideally would be the most effective facilitator, if health care professionals were to think there was a point in doing ACP.

“It is not enough to say it is the patient's healthcare service and turn around and go. What is really needed is to say that you decide in the health care system that the patient's wishes are on the same level as treatment options, for instance. That it is obligatory that you cannot give treatment without the patient's participation. There must be a cultural change and an anchoring in the system” (Key person).

Another way of explaining this was that ACP must have the opportunity to accept what the patient says. As things are now, a physician would be placed in a hypocritical position allegedly sitting down to listen to the patient, when the patient’s opinion ultimately wouldn’t change the decision.

“It's a little strange to ask the patient: what do you think? Because we are sitting with the answer anyway.” (Key person).

### Professional and public education

Deciding that health professionals are responsible for engaging and including this in the education was regarded as the most important facilitator to counteract the barrier of who is responsible. However, several participants also perceived that it would be just as important to educate the public to facilitate engagement, also as a way of changing the culture.

“It is in my opinion part of society or culture to have participation, to have something to say, so I think one is mature and ready for it, and I think that is what you will see in the future” (Chief physician).

## Discussion

This paper describes a qualitative study investigating the perceptions of 40 health care professionals regarding barriers and facilitators to implementing ACP in a context where ACP has not been done systematically before. Taken together with previous literature, our findings highlight many of the known barriers, but new barriers and facilitators were also identified. This knowledge can inform implementation strategies in clinical contexts where ACP is pioneering work.

The lack of time stood out as a very clear pattern echoing findings from previous literature [12, 14, 33, 34], not the least the lack of physical space to have such discussions in hospitals [35]. Discordant documentation and variance in documentation systems [36, 37] were also found in this material, particularly during care transitions [20]. Recognition and support from leadership [38, 39], as well as clinicians’ discomfort and reluctance to discuss death and end of life issue [14, 19, 20, 34], the desire to preserve normalcy [36], uphold hope [12], and a curative focus were all pointed out as barriers. [35]. The difficulty getting the timing right [13, 14, 40], and the lack of knowledge as well as inadequate training [9, 14], and unclear responsibility regarding who should initiate [20], are also known barriers supported in this material.

The overall barrier seems to be the culture, a large obstacle that will take time to change, and that will need to be tackled not only through education, but also practical training. A pattern throughout the material is the worry that ACP could harm the patient, inherently going against health professionals’ core values and their sense of duty to protect their patients. On the other hand, the participants explained that the conversations that did take place mostly represented positive experiences for patients, a finding consistent with previous literature [41, 42].

The reluctance to talk about end of life seemingly further exacerbates the problem and could add to the misconception of ACP as harmful, because it inevitably introduces death. In this study the interviewer tried to enlarge the focus beyond end of life, but the theme of death was always lurking behind, and most participants considered ACP under this narrower scope. In the large implementation project a good strategy would be to clarify that ACP is not only related to end of life, and train professionals in engaging in ACP not in the face of a crisis or shortly before dying.

Related to the association between ACP and death was the reluctance to document patients’ preferences. This concerns a much deeper question regarding the role of the health professional. There was an underlying interpretation that prognostics, diagnostics, assessment and treatment are the main tasks that could be summed up as something biomedical, as opposed to something humanistic, as in just showing compassion without applying the possibilities of medical treatment. The material reflects a fairly black-white assumption regarding interfering medically as opposed to planning for death, with little reflection or practical experience with what would be the gray-zone in between. But ACP could be a way of exploring and opening up that gray-zone and learning new ways to approach complex decisions. Rectifying the misconception that ACP is just a death panel-sign, but rather a process supporting patient autonomy and promoting patient participation is a very important facilitator. This is something that relates to culture and education, but it should also be made clear in the policy and in the law. At the policy level the most important barrier reflected in this study is the discrepancy between the shift towards autonomy and that patients’ preferences are not legally binding. Implementing ACP in such health care systems, would also have to start by taking even one step further: establishing that patients’ preferences count in medical decisions and decisions about care.

Concerning the strength and the limitations of this study, a strength is the approach on a larger scale, focusing on how ACP can be implemented from the different perspectives of the persons who will be using the intervention across levels. Another strength is that the exploration of barriers has been conducted in a context where much discretion is still given to the physician, potentially counter to the autonomy of the patient. Hence these findings can provide new insight for systems where patients’ wishes are not legally binding, albeit patient participation is stated as the norm.

A weakness of this study is that the policy level has not been fully explored by interviewing stakeholders working within government and with the legislation. This will be done in a separate paper.

Another weakness is the lack of triangulation of methods. Participant observation would be a more valid way to describe what that the participants do, if they at all do elements of ACP. Interestingly, a Norwegian study from nursing homes has been conducted with this triangulation of methods and showed a discrepancy between what people say they do and observations that identified a number of challenges inherent in the way ACP was practiced [43].

Finally, a weakness is the lack of the patient perspective as the counterpart stakeholder perspective. What the receiver of ACP perceives as barriers but also how and when ACP is best done cannot be answered without their point of view. This perspective will elaborated in a separate paper, as well as the perspective of the next of kin.

## Conclusion

Progressively changing the education and culture are major efforts that need to be handled in the implementation of ACP, particularly for unfamiliarized contexts. Examples in Europe are Norway, but also Denmark, Sweden, Greece, Italy and Bulgaria [44]. The results of this study reflect that correcting the misconception that ACP is only about death can facilitate early engagement and prevent missed opportunities. The type of conversations reported as ACP by participants, have the nature of a clarification of treatment typically in the face of a crisis or shortly before dying, which is not ACP, and also introduces the idea that ACP could be used as a way of approaching the limitation of treatment, which is very problematic. Providing practical training for health professionals, particularly regarding on how to start ACP, are important facilitators at the clinical level. This is reported by experienced participants as tuning into where the individual patient is, and trying to achieve a common realistic image of the patient's health status to begin with, to increase early engagement and a good progression forward. These steps are also reflected in guidelines [32]. At the institutional level, standardizing how to document and communicate ACP across levels are reported as the most important facilitators, particularly to reduce overtreatment. At the policy level, deciding on and including in the education that health professionals are responsible for initiating, are important facilitators to reduce uncertainty on who should initiate. Finally, developing policies and legislation on patient participation are important steps to go forward with ACP in the services, thus laying the groundwork for better involvement of older patients and their families.

## Supplementary Information

**Additional file 1.** [45–47].

**Additional file 2.**

## Data Availability

The dataset used and/or analyzed during the current study is available from the corresponding author on reasonable request, and upon consent from the relevant participants.

## Abbreviations

AD: Advance directive
ACP: Advance care planning
MD: Medical doctor
GP: General practitioner

## References

[1] Aghabarary M, Dehghan NN (2016). Medical futility and its challenges: a review study. J Med Ethics Hist Med.

[2] Health law - Patients' rights. Britannica.

[3] Beauchamp TL, Childress JF. Principles of biomedical ethics. 8th ed2019.

[4] Sokol LL, Young MJ, Paparian J, Kluger BM, Lum HD, Besbris J (2019). Advance care planning in Parkinson’s disease: ethical challenges and future directions. Parkinson's Disease.

[5] Sudore RL, Lum HD, You JJ, Hanson LC, Meier DE, Pantilat SZ (2017). Defining advance care planning for adults: a consensus definition from a multidisciplinary delphi panel. J Pain Symptom Manage.

[6] Motley M (2013). Improving patient-centered care through advance care planning: three-quarters of patients at the end of life cannot participate in medical decision making. PAs can play key roles in improving the quality of care. JAAPA.

[7] Sabatino CP (2010). The evolution of health care advance planning law and policy. Milbank Q.

[8] Morrison RS, Meier DE, Arnold RM (2021). What's wrong with advance care planning?. JAMA.

[9] Jimenez G, Tan WS, Virk AK, Low CK, Car J, Ho AHY (2018). Overview of systematic reviews of advance care planning: summary of evidence and global lessons. J Pain Symptom Manage.

[10] McMahan RD, Tellez I, Sudore RL (2021). Deconstructing the complexities of advance care planning outcomes: what do we know and where do we go? a scoping review. J Am Geriatr Soc.

[11] Malhotra C, Shafiq M, Batcagan-Abueg APM (2022). What is the evidence for efficacy of advance care planning in improving patient outcomes? a systematic review of randomised controlled trials. BMJ Open.

[12] Sharp T, Moran E, Kuhn I, Barclay S (2013). Do the elderly have a voice? advance care planning discussions with frail and older individuals: a systematic literature review and narrative synthesis. Br J Gen Pract.

[13] Glaudemans JJ, Moll van Charante EP, Willems DL (2015). Advance care planning in primary care, only for severely ill patients? a structured review. Fam Pract.

[14] Fien S, Plunkett E, Fien C, Greenaway S, Heyland DK, Clark J (2021). Challenges and facilitators in delivering optimal care at the End of Life for older patients: a scoping review on the clinicians' perspective. Aging Clin Exp Res.

[15] Gjerberg E, Lillemoen L, Weaver K, Pedersen R, Førde R (2017). Advance care planning in Norwegian nursing homes. Journal of the Norwegian Medical Association.

[16] Klomstad K, Pedersen R, Førde R, Romøren M (2018). Involvement in decisions about intravenous treatment for nursing home patients: nursing homes versus hospital wards. BMC Med Ethics.

[17] Hofacker S, Naalsund P, Iversen GS, Rosland JH (2010). Emergency admissions from nursing homes to hospital at the end of life. Tidsskr Nor Laegeforen.

[18] Hui D, Nooruddin Z, Didwaniya N, Dev R, De La Cruz M, Kim SH (2014). Concepts and definitions for "actively dying," "end of life," "terminally ill," "terminal care," and "transition of care": a systematic review. J Pain Symptom Manage.

[19] Lund S, Richardson A, May C (2015). Barriers to advance care planning at the end of life: an explanatory systematic review of implementation studies. PLoS ONE.

[20] Combes S, Nicholson CJ, Gillett K, Norton C (2019). Implementing advance care planning with community-dwelling frail elders requires a system-wide approach: an integrative review applying a behaviour change model. Palliat Med.

[21] Wilkinson LA, Goldstein S, Naglieri JA (2011). Systems Theory. Encyclopedia of Child Behavior and Development.

[22] Norwegian Law, Patients' Rights Act. 1999.

[23] Kaasa. Paa Liv og Dod [Palliation for the severely ill and dying] NOU 2017:16 [cited 2022 28.6.22]. Available from: https://www.regjeringen.no/contentassets/995cf4e2d4594094b48551eb381c533e/nou-2017-16-pa-liv-og-dod.pdf.

[24] Norwegian Directorate of Health. Decision-making processes in the limitation of lifeprolonging treatment 2013 [cited 2022 29.6.22]. Available from: https://www.helsedirektoratet.no/veiledere/beslutningsprosesser-ved-begrensning-av-livsforlengende-behandling/Decision-making%20processes%20in%20the%20limitation%20of%20life-prolonging%20treatment.pdf/_/attachment/inline/fcaec913-6115-485a-831d-10ab57c1b7ef:31ae370fb2b675b6d56f042ed36dc9ca54569632/Decision-making%20processes%20in%20the%20limitation%20of%20life-prolonging%20treatment.pdf.

[25] Sudore RL, Heyland DK, Lum HD, Rietjens JAC, Korfage IJ, Ritchie CS (2018). Outcomes that define successful advance care planning: a Delphi panel consensus. J Pain Symptom Manage.

[26] Rietjens JAC, Sudore RL, Connolly M, van Delden JJ, Drickamer MA, Droger M (2017). Definition and recommendations for advance care planning: an international consensus supported by the European Association for Palliative Care. Lancet Oncol.

[27] Tong A, Sainsbury P, Craig J (2007). Consolidated criteria for reporting qualitative research (COREQ): a 32-item checklist for interviews and focus groups. Int J Qual Health Care.

[28] Moen K, Middelthon AL. Chapter 10. Qualitative Research Methods. Laake, Breien, Olsen (esd) Research in Medical and Biological Science From Planning and Preparation to Grant Application and Publication: Elsevier; 2015. p. p 331–73.

[29] Gilje N. Hermeneutics – theory and method (In Norwegian). Järvinen M, Mik-Meyer N, editors: Hans Reitzels forlag; 2017.

[30] Braun V, Clarke V (2006). Using thematic analysis in psychology. Qual Res Psychol.

[31] Braun V, Clarke V (2022). Conceptual and design thinking for thematic analysis.

[32] Detering, Silveira. Facilitating advance care planning discussion (UpToDate) [cited 2020 1.2.2020]. Available from: https://www.uptodate.com/contents/advance-care-planning-and-advance-directives?search=advance%20care%20planning&source=search_result&selectedTitle=1~150&usage_type=default&display_rank=1#H2094995

[33] Carter C, Leanza F, Mohammed S, Upshur REG, Kontos P (2021). A rapid scoping review of end-of-life conversations with frail older adults in Canada. Can Fam Physician.

[34] Ke LS, Huang X, O'Connor M, Lee S (2015). Nurses' views regarding implementing advance care planning for older people: a systematic review and synthesis of qualitative studies. J Clin Nurs.

[35] Threapleton DE, Chung RY, Wong SYS, Wong ELY, Kiang N, Chau PYK (2017). Care toward the end of life in older populations and its implementation facilitators and barriers: a scoping review. J Am Med Dir Assoc.

[36] Frechman E, Dietrich MS, Walden RL, Maxwell CA (2020). Exploring the uptake of advance care planning in older adults: an integrative review. J Pain Symptom Manage.

[37] Ahluwalia SC, Bekelman DB, Huynh AK, Prendergast TJ, Shreve S, Lorenz KA (2015). Barriers and strategies to an iterative model of advance care planning communication. Am J Hosp Palliat Care.

[38] Lum HD, Sudore RL, Bekelman DB (2015). Advance care planning in the elderly. Med Clin North Am.

[39] E Gjerberg LL, K Weaver, R Pedersen, R Forde Advance care planning in Norwegian nursing homes. Tidsskrift for den Norske Laegeforening. 2010(130):1721–4. 10.4045/tidsskr.16.0284 28332797

[40] Ryan T, Amen KM, McKeown J (2017). The advance care planning experiences of people with dementia, family caregivers and professionals: a synthesis of the qualitative literature. Ann Palliat Med.

[41] Janssen DJ, Engelberg RA, Wouters EF, Curtis JR (2012). Advance care planning for patients with COPD: past, present and future. Patient Educ Couns.

[42] Davison SN, Simpson C (2006). Hope and advance care planning in patients with end stage renal disease: qualitative interview study. BMJ.

[43] Thoresen L, Ahlzén R, Solbrække KN (2016). Advance care planning in Norwegian nursing homes-Who is it for?. J Aging Stud.

[44] Simón P (2011). Use of advance care planning – a European perspective. BMJ Support Palliat Care.

[45] Aw D, Hayhoe B, Smajdor A, Bowker LK, Conroy SP, Myint PK (2012). Advance care planning and the older patient. QJM.

[46] Bernacki RE, Block SD (2014). Communication about serious illness care goals: a review and synthesis of best practices. JAMA Intern Med.

[47] Rigby MJ, Wetterneck TB, Lange GM (2022). Controversies about advance care planning. JAMA.

