# Supplementary material for: Barriers and facilitators to implementing advance care planning in naïve contexts - where to look when plowing new terrain?
Source: BMC Geriatr. 2023 Jun 23;23:387. doi: 10.1186/s12877-023-04060-4 (PMC10290291; doi:10.1186/s12877-023-04060-4)
Supplement: Supplementary file 1 — Additional file 1. [45–47]. [file 12877_2023_4060_MOESM1_ESM.docx]

**Additional file 1**

**Overview of known barriers and facilitators for ACP and end-of-life-conversations at the clinical and organizational level**

**Barriers at the clinical level**

Prognostication and diagnosing dying (9, 19, 20). Getting the timing right is difficult (13, 14, 21), especially for patients with dementia (9, 20) which gives a lack of preparedness among patients and carers (9).

Family discord or family disagreements (9, 12, 14, 19, 22, 23), to the point that families may pressurize patients into accepting more aggressive forms of care (23) and unacceptable coercion may be difficult to judge (45).

Discussing dying and death (14, 19, 20, 22), the desire to preserve normalcy (25), uphold hope (12), and a curative focus (23).

Clinicians’ discomfort and reluctance to talk about existential issues (46). Discomfort and reluctancy to discuss ACP with patients because they thought end-of-life conversations were difficult (22, 45).

Lack of knowledge (23) and inadequate training pose as a lack of communication skills and preparedness (9, 14).

Paternalism (19).

Fragmentation and lack of trusting relationship and continuity between patients and clinicians (9, 14, 20), and unclear responsibility (19).

**Barriers at the organizational/institutional level**

Time and resource restrictions for doctors and nurses (12, 14, 20, 22), such as lack of physical space in hospitals to have family visits/discussions (23).

Lack of public dissemination on ACP (22).

Discordant documentation and variance in documentation systems (25, 27), which may in worst case go against patient’s wishes (45).

ACP cannot match the multiple scenarios that can take place in a written record (21, 45).

Administrative barriers like off-putting paperwork (20), the lack of leadership and work flow processes (28), specific reimbursement for ACP counseling (22, 28), and appropriate billing codes (19).

Variation in approaches to ACP (27, 28). Policies and guidelines regarding ACP responsibility were thought to be unclear (19), e.g. whether ACP is a means of reducing expenditure of health-care resources (45).

**Facilitators at the clinical level**

Improved communication skills through education and training (14, 19, 22), especially for undergraduate doctors (14) and nurses (22) including what/how to communicate and being more direct and honest regarding prognosis and anticipated outcomes (19).

Supervision and reflective discussions was suggested as solutions to dealing with the psychological impact (14).

Personal ability related to knowledge and skills (19).

Recognizing and acting on triggers including recognizing poor prognostic indicators, transitions, such as admission to homecare services, and environment, particularly living in long-term care (19).

Having informal conversations (21). Conversations should be ‘… focused and brief …’, use open questions such as ‘what things are most important to you, now and in the future?’ and be held in conducive environments (19).

Early engagement for older adults living with frailty, prior to potential physical or cognitive deterioration and reassess (19).

Focusing on living well now rather than planning for dying and death (19).

Using hypothetical scenarios as a strategy to support older adults living with frailty clarifying their views and beliefs regarding end-of-life wishes (19).

Recognizing the impact of relationality, e.g. how ACP decisions may affect others than the patients themselves (19).

Continuity in relations by developing trusting relationships (19, 21).

**Facilitators at the organizational/institutional level**

A cross sectoral, multidisciplinary approach, with the overall recommendation that ACP became ‘… woven into the fabric …’ of everyday practice ‘… as normal as discussing smoking cessation’ (19).

Recognition and support from leaders and managers to facilitate ACP, especially ensuring adequate resourcing such as appropriate staffing (15, 47), finances, education and common documentation (19, 27).

Establishing task forces, interest groups, lobbying and supporting educational efforts, also at the policy level (14).

Integrated and comprehensive system-wide models including developing and maintaining cross-sectoral relationships, ensuring key people, particularly families, were available, enabling documentation access, particularly during care transitions, and community-wide support and education (19).

Access to clear records e.g. about residents’ resuscitation status, preferred place of death and arrangements post-death (14). Valid/accurate prognostic tools to reduce uncertainty (14, 23).
